# Transposable elements as key regulators of placental development

**DOI:** 10.18699/vjgb-25-73

**Published:** 2025-09

**Authors:** M.A. Zhilkina, E.N. Tolmacheva, S.A. Vasilyev

**Affiliations:** Research Institute of Medical Genetics, Tomsk National Research Medical Center of the Russian Academy of Sciences, Tomsk, Russia; Research Institute of Medical Genetics, Tomsk National Research Medical Center of the Russian Academy of Sciences, Tomsk, Russia; Research Institute of Medical Genetics, Tomsk National Research Medical Center of the Russian Academy of Sciences, Tomsk, Russia

**Keywords:** transposable elements, retrotransposons, retroviruses, placenta development, мобильные элементы, ретротранспозоны, ретровирусы, развитие плаценты

## Abstract

Transposable elements (TEs), comprising over one-third of the human genome, play a crucial role in its evolution, serving as a significant source of regulatory sequences. Under normal circumstances, their activity is tightly controlled by DNA methylation mechanisms; however, the effectiveness of this suppression varies substantially across tissues. The placenta, characterized by global hypomethylation, represents a unique environment where retroviruses and retrotransposons, typically silenced in somatic cells, gain the opportunity for activation. This distinct epigenetic landscape of the placenta allows transposons to participate in the regulation of genomic activity, influencing processes ranging from early embryogenesis to postnatal development. DNA hypomethylation in the placenta not only promotes TE mobilization, but also opens the possibility of using their components as independent genes and regulatory elements – promoters, enhancers, and other functional modules. These elements are involved in key aspects of placental development, including syncytiotrophoblast formation, extravillous trophoblast invasion, spiral artery remodeling, and endometrial decidualization. Importantly, TEs can serve as sources of alternative promoters for neighboring genes, and ancient mammalian transposons contain multiple transcription factor binding sites, enabling coordinated regulation of genes sharing a common function. Despite the growing interest in the role of transposable elements in placental development and function, many questions remain unanswered. In particular, the mechanisms of non-long terminal repeat (non-LTR) retrotransposon function during pregnancy remain poorly understood. A deep understanding of these processes is necessary to elucidate regulatory disorders in the placenta associated with major obstetric syndromes. This review examines the contribution of transposable elements to the functioning of the human genome, particularly their impact on gene expression, in the context of pregnancy and placental development

## Abstract (ru)

Мобильные элементы (transposable elements, TE), составляющие свыше трети человеческого генома, играют ключевую роль в его эволюции, выступая важным источником регуляторных последовательностей. В норме их активность жестко контролируется механизмами метилирования ДНК, однако эффективность такого подавления существенно различается между тканями. Плацента, отличающаяся глобальным гипометилированием, представляет собой уникальную среду, где ретровирусы и ретротранспозоны, обычно молчащие в соматических клетках, получают возможность активации. Этот особый эпигенетический ландшафт плаценты позволяет транспозонам участвовать в регуляции геномной активности, оказывая влияние на процессы, протекающие от раннего эмбриогенеза до постнатального развития. Гипометилирование ДНК в плаценте не только способствует мобилизации TE, но и открывает возможность использования их компонентов в качестве самостоятельных генов и регуляторных элементов – промоторов, энхансеров и других функциональных модулей. Эти элементы вовлечены в ключевые аспекты плацентарного развития, включая формирование синцитиотрофобласта, инвазию вневорсинчатого трофобласта, ремоделирование спиральных артерий и децидуализацию эндометрия. Важно отметить, что TE могут служить источниками альтернативных промоторов для соседних генов, а древние транспозоны млекопитающих содержат множественные сайты связывания транскрипционных факторов, обеспечивая скоординированную регуляцию генов, объединенных общей функцией. Несмотря на растущий интерес к роли мобильных элементов в развитии и функционировании плаценты, многие вопросы остаются без ответа. В частности, малоизученными продолжают быть механизмы функционирования в ходе беременности ретротранспозонов, не содержащих длинных концевых повторов (non-LTR ретротранспозонов). Глубокое понимание этих процессов необходимо для прояснения нарушений регуляции в плаценте при больших акушерских синдромах. В данном обзоре рассматривается вклад мобильных элементов в функционирование генома человека, в частности их влияние на экспрессию генов, в контексте беременности и развития плаценты

## Introduction

Approximately 40 % of the mammalian genome is comprised of mobile genetic elements called "transposons" (TEs) (Chesnokova et al., 2022). At first glance, such an abundance of TEs in mammalian genomes seems paradoxical, given the potential risks associated with uncontrolled transposition (Doolittle, Sapienza, 1980). However, this coexistence reflects an ongoing evolutionary arms race between TEs and their hosts, resulting in a dynamic equilibrium. Although most mammalian TEs have been inactivated through mutations or transcriptional/ post-transcriptional silencing, there are exceptions. Some TE/host interactions, initially driven by the need for TE replication, can be repurposed to perform important functions in host development or physiology.

In recent decades, it has become clear that such adaptation of mobile genetic element sequences to perform new functions in the host genome is a crucial step in their evolution. J. Brosius and S.J. Gould (1992) made a significant contribution to understanding this process, challenging the view of mobile genetic elements solely as “junk DNA” and proposing that TEs should be considered a source of evolutionary innovation through the mechanism of exaptation – the repurposing of existing genetic elements to perform new functions. It is important to note that while adaptation involves the refinement of features under the direct selection for their current function, exaptation describes the use of pre-existing traits for entirely new purposes (Brosius, Gould, 1992). As such, mobile genetic elements (such as transposons and retrotransposons) can take on biologically significant roles in gene regulation, formation of new functional elements of the genome or its structure (Chuong et al., 2016).

The reproductive strategy of placental mammals, characterized by intrauterine development and prolonged lactation, requires significant energetic and metabolic expenditures on the part of the maternal organism (Hamilton, Boyd, 1960). Under such substantial maternal costs, natural selection predictably favors the development of mechanisms for early elimination of non-viable embryos in the early stages of ontogenesis. From this perspective, the matter of the preservation of mobile elements in the genome, despite their potentially destructive effects and the strong action of selection, is of scientific interest

The evolutionary persistence of TEs can be explained by their strategic integration into key processes that determine organism viability at critical stages of development. These fundamental processes include an activation of the embryonic genome, a successful embryo implantation, and placentation. The effectiveness of this strategy is supported by the large-scale invasion of TEs into mammalian genomes.

The features of epigenetic regulation in the placenta, such as global DNA hypomethylation and the presence of partially methylated domains of extended genomic regions with intermediate levels of methylation (Novakovic, Saffery, 2013), provide unique conditions for the activation of endogenous retroviruses and retrotransposons that are repressed in most somatic tissues (Honda, 2016). The brevity of existence and temporary nature of placenta as an organ further explains the specificity of its epigenome organization.

This review attempts to systematize current data on the functional significance of mobile elements in placental development and function.

## Transposable elements in mammalian genomes

Mobile genetic elements are highly abundant in mammalian genomes. While previously considered “junk DNA”, their significant influence on host genome function is now a wellestablished fact. According to current data, approximately 50 % of the human genome is comprised of retrotransposons and DNA transposons (de Koning et al., 2011).

From the perspective of molecular transposition mechanisms, all transposable elements (TEs) are divided into two main classes (Wicker et al., 2007). The first class groups the elements called “retrotransposons”. For movement, these elements utilize an RNA intermediate followed by reverse transcription via a “copy-and-paste” mechanism while preserving the original sequence intact (Mustafin, 2018). The second class is represented by DNA transposons, which transpose genes without RNA involvement via a “cut-and-paste” mechanism (TIR and Cryptons) or through replicative transposition (Helitrons and Mavericks) (Mustafin, 2018). Retrotransposons, in turn, are classified into five orders based on their molecular organization, transposition mechanisms, and reverse transcriptase phylogeny: endogenous retroviruses (ERVs) with long terminal repeats (LTRretrotransposons), LINE and SINE elements, DIRS-like elements, and Penelope-like elements (Wicker et al., 2007).

Typical ERVs contain three conserved coding domains (gag, env, pol) and are flanked by identical long terminal repeats (LTRs) on both sides (see the Figure). However, over the course of vertebrate evolution, most ERVs have acquired multiple mutations, resulting in the loss of their ability to fully express viral proteins (Johnson, 2019). The human-specific group of LTR-containing retrotransposons is commonly referred to as HERVs (human endogenous retroviruses).

**Fig. 1.**
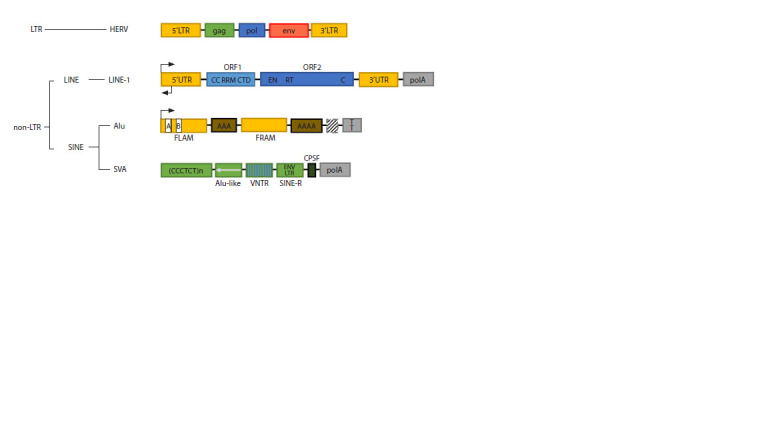
LTR-containing and non-LTR retrotransposons in the human genome. For HERV retrotransposons: long terminal repeat (LTR) (yellow blocks), and gag (green block), env (red block), pol (blue block) encoding domains. For LINE-1 retrotransposon: untranslated regions (UTR) (yellow blocks); sense and antisense internal promoters (black arrows); ORF1 includes a coiled-coil (CC) domain, RNA recognition motif (RRM) and C-terminal domain (CTD); ORF2 includes endonuclease (EN), reverse transcriptase (RT) and cysteine-rich (C) domains; poly(A) tail (polA follows 3’ UTR). For Alu: FLAM (free left Alu monomer); FRAM (free right Alu monomer); RNA polymerase III transcription start site (black arrow) and conserved cis-acting sequences required for transcription (white blocks A and B in the left Alu monomer); adenosine-rich fragment (brown block AAA between the left and right Alu monomers); terminal poly(A) tail (brown block AAAA); flanking genomic DNA of variable size (hatched gray block), followed by the pol III RNA termination signal (gray block TT). For human SVA: CCCTCT hexameric repeat; inverted Alu-like repeat (green block with a reverse arrow); GC-rich VNTR (hatched green block); SINE-R sequence homologous with HERV-K10 (ENV and LTR regions); cleavage and polyadenylation specificity factor (CPSF) binding site; terminal poly(A) tail (polA) (as per Lee et al., 2024).

Mobile genetic elements lacking long terminal repeats (non-LTR) are primarily represented by two classes: long interspersed nuclear elements (LINEs) and short interspersed nuclear elements (SINEs). LINE elements have a length of several thousand nucleotide pairs, whereas the size of SINEs usually does not exceed 600 base pairs (Kramerov, Vassetzky, 2011; Bourque, 2018). The fundamental difference between these groups lies in their transcription mechanisms: LINEs, like LTR-retrotransposons, are expressed by RNA polymerase II, while most SINEs are transcribed with the involvement of RNA polymerase III (Kramerov, Vassetzky, 2011).

SINE elements exhibit exceptionally high abundance in mammalian genomes, exceeding 100,000 copies. They replicate employing a retrotransposition mechanism based on the “copy-and-paste” sequential transcription into RNA, reverse transcription to form cDNA, and integration into new genomic loci. This process is entirely dependent on the enzymatic machinery encoded by LINE elements. In the human genome, the most prevalent SINE family is Alu – 300-nucleotide sequences that evolved from 7SL RNA (Lee et al., 2024).

LINE-1 elements, constituting approximately 17 % of the human genome (Chesnokova et al., 2022), have a complex structure. Full-length functional copies, of which there are approximately one thousand, contain untranslated regions (UTRs) necessary for transpositional activity. These elements include a 5ʹ-UTR with a unique bidirectional promoter, two open reading frames (ORF1 and ORF2) encoding ORF1p and ORF2p proteins, and a 3ʹ-UTR with a polyadenylation signal (see the Figure). Of particular interest is the organization of the LINE-1 promoter region, containing both a sense promoter regulating the expression of retrotransposition proteins and an antisense promoter (ASP) (Lee et al., 2024).

Regulation of retrotransposon activity in mammalian somatic cells is critical for maintaining genomic stability. Numerous studies confirm the key role of epigenetic mechanisms, particularly DNA methylation, in suppressing the potentially hazardous transpositional activity of these elements (Slotkin, Martienssen, 2007). This control mechanism act as an important protective barrier, preventing the development of genomic disorders and associated pathological conditions

## Unique epigenetic landscape of the placenta

The placenta is characterized by global DNA hypomethylation, which distinguishes its epigenetic profile from somatic tissues (Ehrlich et al., 1982). The average level of 5-methylcytosine in human placental tissue is 2.5–3 %, whereas in umbilical cord blood it reaches ~4 % (Price et al., 2012). The given epigenetic status is a key factor in regulating expression of genes, controlling placental growth and trophoblast functional activity (Robinson, Price, 2015).

The premise of placental hypomethylation is epigenetic reprogramming, a key feature of which in the zygote and embryo at the preimplantation stage of development is the loss of DNA methylation, such that the late morula/early blastocyst exhibits the lowest level of DNA methylation compared to any other period of ontogenesis. Subsequent de novo methylation in the inner cell mass is accompanied by TE repression, while placenta-forming trophectoderm cells maintain a hypomethylated state of these elements (Price et al., 2012).

Although the functional role of the reduced level of genome methylation observed in the placenta is still not fully understood, studies show that it can activate the expression of mobile elements that is normally suppressed in other tissues (Macaulay et al., 2011). DNA methylation of HERV families in the placenta exhibits widely varying, but on average reduced levels compared to embryonic and adult tissues (Reiss et al., 2007). In contrast, the average DNA methylation index of Alu is similar in placental and fetal tissues (Price et al., 2012; Rondinone et al., 2021), and DNA methylation of the LINE-1 retrotransposon is reduced and more variable in the placenta compared to fetal tissues.

However, a decrease in TE methylation levels does not always lead to an increase in their transcriptional activity. For example, in a recent study, S. Lanciano et al. found that only a small number of copies of young L1s are activated upon decreased DNA methylation in the genome, whereas most hypomethylated L1 loci unexpectedly remain silent (Lanciano et al., 2024). The promoters of young active L1 elements are hypomethylated in human embryonic stem cells compared to differentiated cells, which partially explains their higher level of expression

Hypermethylation of LINE-1 in the placenta has also been reported in some pregnancy pathologies. Hydatidiform mole is one cause of pregnancy loss and the most common type of gestational trophoblastic disease. In patients with hydatidiform mole, a twofold increase in LINE-1 methylation levels was observed throughout placental development and differentiation, whereas the level of overall genome methylation and other repeats remained the same in this pathology (Lou et al., 2020). In spontaneous abortions with aneuploidy, increased LINE-1 methylation was observed in extraembryonic tissues (Vasilyev et al., 2021). However, at the same time, LINE-1 is hypomethylated in extraembryonic tissues of spontaneous abortuses with a normal karyotype, which can lead to enhanced LINE-1 activation and subsequent mutational insertions (Lou et al., 2020).

An example of the influence of hypomethylation on the activity of mobile elements in the placenta is the hypomethylation of the AluY retrotransposon in the KCNH5 locus. A differentially methylated region in the promoter region and first exon of transcript 1a of the KCNH5 gene is of retrotransposon origin: 147 bp of the promoter and 162 bp of the exon evolved from a SINE-element of the AluY family. This element, which first appeared in the primate genome about 25–30 million years ago, has been preserved only in humans, great apes, and Old World monkeys, indicating its recent (on an evolutionary scale) integration. Hypomethylation of AluY in the placenta correlates with activation of an alternative KCNH5 transcript, demonstrating how epigenetic modification of mobile elements can participate in tissue-specific gene regulation (Macaulay et al., 2011).

## Functional exaptation of mobile genetic elements in the placenta

Low levels of DNA methylation in the placenta have facilitated the use of TE parts as functional regulatory sequences. In particular, TEs have been integrated into placenta-specific enhancers, alternative promoters, and other cis-regulatory elements, contributing to the evolutionary diversification of placental functions (Hoyt et al., 2022).TE derivatives play an important role in various processes, including altering splicing patterns, enhancing recombination, forming enhancer and silencer regions, utilizing alternative promoters, and gene neofunctionalization (Brosius, 1999). The regulatory activity of TEs is apparent as early as the blastocyst stage and is maintained throughout mammalian prenatal development, including in the placenta. Certain integrated retroviral sequences have evolved into critically important regulatory elements, modulating the expression of neighboring genes or even forming novel gene loci (Johnson, 2019).

The genes ERVW-1 (syncytin-1) and ERVFRD-1 (syncytin- 2) are classical examples of HERV elements that have undergone exaptation, acquiring placenta-specific functions (Macaulay et al., 2011). They have retained the ability to encode envelope (env) proteins, which typically mediate viral entry into cells (Nelson et al., 2003). However, in the placenta, these proteins have acquired a novel physiological function: they mediate the differentiation and fusion of cytotrophoblast cells, leading to the formation of the multinucleated syncytiotrophoblast (Pötgens et al., 2002).The syncytin family, derived from HERVs, is a unique group of fusogenic proteins that play a crucial role in placental morphogenesis. Experimental data indicate that the surface SU domain of these proteins is essential for cell fusion, as evidenced by its inhibition with specific antibodies (Shimode, 2023).

In addition to cell fusion, syncytin-1 regulates critical functions such as proliferation and antiviral responses in trophoblast stem cells (West et al., 2022). Syncytin-2 contains a typical retroviral immunosuppressive env domain (Mangeney et al., 2007). Its expression in human cytotrophoblast cells suggests the involvement of this protein in establishing immunological tolerance during pregnancy, potentially through suppression of the maternal immune response to the fetus. Thus, former viral envelope proteins have been adapted to perform entirely new functions that are crucial for successful pregnancy

The protein suppressin, the gene of which also originates from an ERV, performs opposing functions by inhibiting cell fusion. Suppressin has been identified in cultured human trophoblast cells and placental tissue samples. Suppressin utilizes ASCT2 as a receptor to inhibit syncytin-1-mediated fusion of cytotrophoblast cells (Sugimoto et al., 2013). In placental development, the balance of syncytin and suppressin gene expression determines the differentiation pathways of trophoblasts. It directs cells either towards fusion, forming the multinucleated syncytiotrophoblast, or towards invasion, forming the invasive trophoblast. Therefore, the regulation of these two HERV-derived genes is critical for normal placental formation and function

The imprinted genes PEG10 (paternally expressed 10) and PEG11/RTL1 (retrotransposon like 1), expressed from the paternal homolog, are also derived from ERVs. PEG10 contains two overlapping open reading frames, the product of one of which features protease activity and plays an important role in the formation of fetal capillaries in mice (Clark et al., 2007). Both PEG10 and PEG11/RTL1 encode proteins that are highly homologous with the group-specific antigen and polymerase proteins of the sushi-ichi retrotransposon of the pufferfish genome, which belongs to the Ty3/ gypsy family (Kim et al., 1994; Song et al., 1994). Functional studies in model organisms have demonstrated the key role of these genes in embryonic development

In PEG10 knockout mice, the labyrinthine and trabecular layers of the chorion are absent, accompanied by early embryonic lethality (Ono et al., 2006). Furthermore, CRISPR-Cas-induced deletion of PEG10 in trophoblast stem cells led to impaired differentiation. Increased expression of the PEG11 gene, or its deficiency, led to late embryonic lethality and neonatal death with damage to placental capillary networks in mice (Sekita et al., 2008; Kitazawa et al., 2017). These data highlight the fundamental significance of ERV-derived genes for ensuring normal placental development and successful pregnancy, demonstrating complex evolutionary mechanisms of exaptation of viral elements to perform critical physiological functions

## Mobile genetic elements as a source of placenta-specific enhancers

TE-related sequences are widespread throughout the human genome, found both within genes and in adjacent regulatory regions. According to Refseq data, 27.4 % of transcribed human DNA sequences have at least one transcript variant with insertions of TE sequences in untranslated regions (van de Lagemaat, 2003). Approximately 45 % of human enhancers are TE-derived (Simonti, 2017).

The function of enhancers is to regulate gene expression through the binding of transcription factors. In placental tissue, a significant prevalence of certain transposon classes is observed among placenta-specific enhancers. LTR-retrotransposons show the highest representation, followed by SINE, LINE, and DNA transposon elements (Sun et al., 2021). In humans, TE-derived enhancers are involved in context-specific gene regulation, including the expression of genes related to pregnancy, early embryonic development, and the formation of innate immunity (Modzelewski et al., 2022).

Human placental enhancers often overlap with specific families of endogenous retroviruses (ERVs), including MER21A, MER41A/B, and MER39B, typically associated with immune responses and placental function (Sun et al., 2021). MER41A/B elements create multiple binding sites for transcription factors, including ones located near the FBN2 gene, which encodes the placenta-specific peptide hormone placentin, stimulating glucose secretion and trophoblast invasion (Yu et al., 2020; Sun et al., 2021). The MER41 family has six subfamilies, including A/B/C/D/E/G (Kojima, 2018). The evolutionary significance of these elements is underscored by their role in the formation of interferon-stimulated cis-regulatory elements that interact with the key transcription factors STAT1 and IRF1 (Schmid, Bucher, 2010; Chuong et al., 2016; Buttler, Chuong, 2022).

Another important example of TE-derived regulatory elements is LTR10A, acting as a powerful enhancer for essential placental genes, including ENG (Frost et al., 2023). The ENG protein plays a significant role in regulating trophoblast differentiation (Mano et al., 2011).Leptin (LEP), encoded by one of the TE-regulated genes, performs multiple functions in early pregnancy. This hormone is involved in regulating implantation, trophoblast invasion, and placental angiogenesis, creating the necessary conditions for normal fetal development (Pérez‐Pérez et al., 2018). In addition to its regulatory function, leptin promotes trophoblast proliferation and inhibits apoptotic processes (Magariños et al., 2007, Pérez‐Pérez et al., 2008). LEP expression in the placenta is controlled by the transposon MER11 (Bi et al., 1997).

Of equal importance is the corticotropin-releasing hormone (CRH) gene, which regulates the duration of pregnancy. Its placental expression is controlled by the primatespecific element THE1B (Dunn-Fletcher et al., 2018).

LTR8B and MER11D elements, which are associated with the PSG gene cluster (Frost et al., 2023), encoding pregnancy-specific glycoproteins, exhibit notable evolutionary patterns. Their distribution among primates correlates with the type of placentation: from 6–24 genes in Old World monkeys to 1–7 genes in New World monkeys and complete absence in lemurs with epitheliochorial placentas (Zimmermann, Kammerer, 2021). Convergent evolution of this cluster in primates and mice (Rudert et al., 1989) suggests its important role in the development of the hemochorial placenta. These results suggest that integration of LTR8B elements prior to the expansion of the PSG cluster in humans was an important step that contributed to high expression of these genes in the trophoblast.The function of PSG genes during pregnancy remains unclear. However, low levels of circulating PSGs are associated with recurrent pregnancy loss, fetal growth restriction, and preeclampsia (Towler et al., 1977; Arnold et al., 1999).

MER61D/E elements employ a specific regulatory mechanism, participating in the formation of binding sites for the transcription factor TP63 (Li et al., 2014). This factor, related to p53 (Riege et al., 2020), supports trophoblast proliferation, preventing premature differentiation. MER61 elements expand the TP63 binding network, participating in cellular stress responses (Su et al., 2015), thus highlighting the multifunctional nature of transposable elements with regards to placental development regulation

## Placenta-specific gene expression from transposable element promoters

Promoters formed from transposons represent an evolutionarily significant mechanism of coordinated gene regulation (Modzelewski et al., 2022). Such mechanisms are particularly important in critical stages of embryonic development, requiring precise temporal and spatial organization of gene expression. Furthermore, transposon-derived regulatory elements enhance the reliability of genetic programs by creating redundancy in transcriptional factor interaction networks.

All orders of retrotransposons and DNA transposons can initiate the formation of chimeric transcripts in mammalian embryos, although their relative activity varies significantly among species and developmental stages (Oomen et al., 2025). The highest concentration of such transcripts is observed in oocytes and at the stage of embryonic genome activation, covering the period from the two-cell stage to the compacted morula stage (8–16 blastomeres). The integration of TE-derived promoter sequences into the host genome creates evolutionary prerequisites for the emergence of new gene expression patterns in various cell types, and also contributes to the generation of shortened or elongated protein isoforms, which ultimately can lead to gene neofunctionalization (Ashley et al., 2018).

Comparative analysis of the transcriptomes of preimplantation embryos of five placental mammal species (mice, pigs, cows, rabbits, and rhesus macaques) revealed species-specific features of transposon-mediated regulation. LTR elements predominate in mouse embryos (59 % of all TE-initiated transcripts), while LINE elements dominate in rabbits (40 %), and SINE elements, in rhesus macaques (42 %). Notably, SINE elements, despite their relatively recent evolutionary origin, demonstrate the ability to form chimeric transcripts in all studied species, although their number varies greatly: from 112 transcripts in cows to 3,910 in rhesus macaques (Oomen et al., 2025). These findings emphasize the important role transposon elements play in regulating early embryonic development in placental mammals

The mechanisms of TE-mediated expression regulation significantly differ. Transposon-derived promoters can either fuse with canonical gene promoters or completely replace them, as well as function as alternative regulatory elements located in various positions relative to the transcription initiation site. LTR elements, which retain promoter activity in both the sense and antisense orientations, are of particular interest (van de Lagemaat et al., 2003).

A classic example of TE-mediated regulation is the CYP19A1 gene, which encodes aromatase P450 and the expression of which in the placenta is controlled by the LTR promoter MER21A (van de Lagemaat et al., 2003). Another significant example is the pleiotrophin (PTN) gene, demonstrating tissue-specific alternative regulation: while one of its transcripts is expressed ubiquitously, including placental tissue, another variant, controlled by the 5ʹ-LTR HERV-E, exhibits strict placenta-specificity (Reiss et al., 2007; Benson et al., 2009).

Another example is the INSL4 gene, encoding a primatespecific peptide of insulin-like hormone and involved in the apoptosis of placental cells. Its expression is controlled by a HERV element, which likely determines the placentaspecific nature of expression in humans and other modern primates (Macaulay et al., 2011).

The LINE-1 antisense promoter can also initiate the formation of chimeric transcripts, in which 5ʹ-antisense LINE-1 sequences are joined to exons of neighboring genes via splicing. However, the functional significance of LINE-1 element activation in the placenta is currently unclear. Previously, using bioinformatics methods, 988 putative LINE-1 chimeric transcripts were identified, with 911 of them being described for the first time (Criscione et al., 2016). Notably, the products of genes specific to neural tissue and placenta predominate among these transcripts, but experimental confirmation of these data has not yet been obtained.

An important step in the establishment and maintenance of pregnancy in many placental mammals is the differentiation (decidualization) of endometrial stromal fibroblasts into decidual stromal cells in response to progesterone. Decidualization triggers extensive reprogramming in the endometrium, leading to dramatic changes in gene expression, recruitment of immunosuppressive immune cells, vascular remodeling, and secretory transformation of uterine glands (Gellersen et al., 2007). In mammals, approximately 13 % of differentially expressed endometrial genes are located within 200 kb of the placental mammal-specific DNA transposon MER20 (Class II TE), which is thought to be involved in regulating key placental genetic networks, including cAMP-dependent signaling pathways in endometrial cells (Lynch et al., 2011). MER20 contains multiple binding sites for various transcription factors, and ancient mammalian transposons in general are enriched in hormone-sensitive regulatory elements that define endometrial cell identity (Lynch et al., 2015).

Furthermore, mapping of functionally active regions of the genome in decidual stromal cells showed that approximately 90 % of open chromatin regions, 58 % of enhancers, and 31 % of promoters overlapped with DNA regions derived from ancient TEs, most of which were specific to mammals or eutherians (Lynch et al., 2015).Thus, the evolutionary domestication of transposable elements in the mammalian genome has led to the formation of a unique regulatory landscape necessary for the development and function of the placenta as an evolutionary novel organ. These changes have affected both the embryonic and maternal components of the placenta, providing complex mechanisms for their interaction.

## Alternative mechanisms of mobile genetic element impact on placental development

In addition to the aforementioned mechanisms of placental development regulation, TEs perform other functions in mammals that, albeit not studied in detail with respect to placentation, play a significant role in early embryonic development, likewise characterized by genome hypomethylation

Of particular interest is transposon-dependent alternative splicing, in which TEs can contain donor or acceptor splice sites, modifying the canonical pre-mRNA processing pathways and contributing to the emergence of new protein isoforms with unique functional properties (Modzelewski et al., 2022).

A vivid example of this phenomenon is the AluY element, which integrated into intron 6 of the TBXT gene in the hominoid ancestor’s genome about 25 million years ago. The interaction of this element with the older AluSx1, located in the reverse orientation, leads to the formation of a hairpin structure in pre-mRNA, which excludes exon 6 from the mature mRNA. The resulting TBXTΔexon6 alternative isoform, specific to hominoids, correlates in time with the loss of a tail in this evolutionary lineage. Experimental expression of this isoform in mice leads to tail development disorders, confirming the key role of this TE-mediated modification in the evolution of primate morphology (Xia et al., 2024).Transposon elements also have a significant impact on chromatin architecture through the formation of binding sites for the CTCF protein, which mediates the formation of topologically associated domains (TADs) (Rao et al., 2017). Approximately 20 % of species-specific TADs contain CTCF sites encoded by species-specific TEs (Choudhary et al., 2020). Although most TAD boundaries are evolutionarily conserved (Vietri et al., 2015), SINE elements show significant enrichment in these regions (Lu et al., 2020), apparently performing a stabilizing function for CTCF-site clusters (Kentepozidou et al., 2020). TEs can also contribute to the establishment of species-specific chromatin loops by introducing new CTCF anchor motifs (Choudhary, 2020).

Notably, some TADs in pluripotent stem cells are formed by an alternative mechanism dependent on the transcription of HERV-H elements (Santoni et al., 2012, Ohnuki et al., 2014). Likewise, in mouse embryos at the 2-cell stage, MERVL elements are not only the main source of promoters for controlling early embryonic development gene expression, but also contribute to the formation of domain boundaries (Kruse et al., 2019). Similar mechanisms may be involved in establishing specific chromatin conformations and in trophoblast stem cells, determining their differentiation potential.

TEs are actively involved in chromatin remodeling processes. It has been shown that the expression of LINE-1 plays an important role in chromatin organization during the activation of the mouse zygotic genome. Prolonged transcriptional activation of LINE-1 or premature transcriptional suppression of LINE-1 in mouse zygotes leads to developmental arrest. At the same time, this effect is not explained by the proteins encoded by LINE-1, but depends on the expression of non-coding RNA LINE-1 (Jachowicz et al., 2017). They act as a nuclear scaffold to recruit the Nucleolin and Kap1 proteins to suppress the Dux/MERVL transcription program at the two-cell stage and maintain the function of the pluripotency gene network in mouse embryonic stem cells (Percharde et al., 2018).

It is also notable that HERV-K elements, which have been relatively recently introduced into the human genome (Belshaw et al., 1999), are actively transcribed during normal human embryogenesis, starting from the eight-cell stage to the preimplantation blastocyst stage. Similar mechanisms may regulate the balance between proliferation and differentiation of trophoblast cells, determining the correct formation of placental structures.

An important evolutionary mechanism is TE-mediated recombination, often occurring between species-specific Alu and LTR elements, which can lead to gene duplication with subsequent neofunctionalization. A classic example is the duplication of the growth hormone gene in catarrhine primates, caused by recombination between Alu elements (Barsh et al., 1983). Growth hormone from duplicated genes is expressed in the placenta and interacts with growth hormone and prolactin receptors in placental tissues (Haig, 2008). This example demonstrates how TE-mediated genomic rearrangements can directly affect placental physiology and evolution.

Thus, numerous studies confirm that the early development of human embryos occurs with the active participation of retroviral and retrotransposon transcripts (Grow et al., 2015), which emphasizes the fundamental role of these elements in the formation and regulation of embryogenesis in mammals. Similar mechanisms likely operate in the placenta, making mobile elements important participants in the formation and function of this unique organ.

## Conclusion

The evolutionary persistence of transposons in mammalian genomes is partly driven by their strategic integration into critical stages of early placental development. It is pivotal that this integration provides TEs with a dual selective advantage: guaranteed vertical transmission via incorporation into genes essential for implantation and placentation (where their elimination leads to embryonic lethality), and access to a unique epigenetic niche. Global hypomethylation and the presence of partially methylated domains in the placenta create an environment permissive to limited retrotransposon activity without catastrophic consequences for the host organism.

Of critical significance is the synergy between the transient nature of the placenta and TE replicative strategies: the ephemeral existence of this organ mitigates the long-term risks of uncontrolled transposition, while simultaneously providing a unique opportunity for functional testing of novel mobile element insertions. Partially methylated domains in the trophoblast genome serve as a molecular platform for exaptation, where potentially beneficial new regulatory mechanisms (such as providing alternative promoters or enhancers) are selectively fixed in the genome. Such dynamics transforms an initially parasitic relationship into a symbiotic one, where TEs are guaranteed replication and transmission, and the host benefits from a source of evolutionary innovation for regulating placental development

## Conflict of interest

The authors declare no conflict of interest.
